# Modulated Interactions Induced by Cyano‐Modified Wide‐Bandgap Small‐Molecule Acceptors Enables High‐Performance Ternary Organic Photovoltaics

**DOI:** 10.1002/advs.202506606

**Published:** 2025-06-20

**Authors:** Yuanyuan Zhang, Shijie Cheng, Meijia Chang, Lei Wang, Huanhuan Gao, Zirui Wang, Guanghao Lu, Shengmin Gan, Xinbo Lv, Jin Wang, Qingqing Sun, Mingjun Niu, Zichao Shen, Zhijun Wu, Cao Yang, Xuying Liu, Lingxian Meng

**Affiliations:** ^1^ School of Materials Science and Engineering Zhengzhou University Zhengzhou 450001 China; ^2^ School of Energy and Chemical Engineering Henan Key Laboratory of Green Building Materials Manufacturing and Intelligent Equipment Luoyang Institute of Science and Technology Luoyang 4710023 China; ^3^ College of New Energy Xi'an Shiyou University Xi'an Shaanxi 710065 China; ^4^ Frontier Institute of Science and Technology Xi'an Jiaotong University Xi'an 710054 China; ^5^ State Key Laboratory of Applied Organic Chemistry (SKLAOC) Key Laboratory of Special Function Materials and Structure Design (MOE) College of Chemistry and Chemical Engineering Lanzhou University Lanzhou 730000 China; ^6^ Institute of Science and Technology for New Energy Xi'an Technological University Xi'an 710021 China

**Keywords:** cyano group, intermolecular interactions, small‐molecule acceptors, surface energy, ternary organic solar cells

## Abstract

The cyano group is extensively employed in the molecular engineering of high‐performance small‐molecule acceptors (SMAs) for organic solar cells (OSCs) to fine‐tune energy levels and optimize molecular packing. To date, the application of cyano group has predominantly been confined to end‐group modification in SMAs, with limited investigation in central unit engineering. Herein, in this work, the role of cyano substitution is systematically investigated in the central unit of SMAs and design a novel cyano‐functionalized wide‐bandgap acceptor UF‐BCN. The introduction of the cyano group significantly enhances the surface energy of the molecule and substantially deepens the highest occupied molecular orbital (HOMO) energy level due to its strong electron‐withdrawing capability, then leading to a blue‐shifted absorption. When introduced as the third component in the D18:BTP‐eC9, UF‐BCN demonstrates complementary light absorption, strong intermolecular interactions, and excellent compatibility with BTP‐eC9 to form a mixed acceptor phase, enabling it to function as an effective morphological modulator within the ternary system. Consequently, the ternary OSC based on D18:BTP‐eC9:UF‐BCN achieves an impressive power conversion efficiency (PCE) of 19.34%. This study underscores the effectiveness of cyano substitution in central unit engineering and highlights its potential for optimizing active layer morphology and enhancing the performance of ternary OSCs.

## Graphical abstract

A novel wide‐bandgap acceptor, UF‐BCN, is designed where cyano substitution significantly enhances surface energy and HOMO levels. When incorporated into a ternary system with D18:BTP‐eC9, UF‐BCN achieves a high‐power conversion efficiency of 19.34% by forming a mixed acceptor phase with strong intermolecular interactions, highlighting the role of the cyano group in central unit engineering of small‐molecule acceptors.

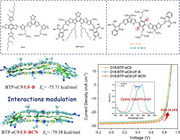

## 1 Introduction

Organic solar cells (OSCs) have emerged as a highly promising technology for next‐generation photovoltaics, offering unique advantages such as low‐cost, lightweight, roll‐to‐roll manufacturing processes and flexibility.^[^ ^1^, ^2^, ^3^, ^4^, ^5^, ^6^, ^7^, ^8^, ^9^ ^]^ In recent years, the substantial advancements in molecular engineering of small molecule acceptors (SMAs), together with the device optimization, have propelled the power conversion efficiencies (PCEs) of OSCs beyond 20%.^[^ ^10^, ^11^, ^12^, ^13^, ^14^, ^15^, ^16^, ^17^, ^18^ ^]^ Nevertheless, despite these remarkable achievements, the inadequate light absorption and energy loss originated from the low charge mobility and dielectric constant of organic materials still impede the further enhancement of the PCEs, then hinder the commercial viability of OSCs.^[^ ^19^, ^20^, ^21^, ^22^ ^]^ To address these challenges, ternary OSCs have emerged as an effective strategy by introducing a third component with complementary absorption.^[^ ^23^, ^24^ ^]^ Such a third component could not only provide complementary absorption but also optimize the morphology of the active layers, demonstrating it a highly compelling approach for achieving higher performance and facilitating the transition from laboratory‐scale research to practical industrial applications.^[^ ^12^, ^25^, ^26^, ^27^, ^28^, ^29^, ^30^, ^31^ ^]^

For the extensively studied ternary OSCs, the selection of the third component is critical for achieving higher PCEs. Currently, in most binary systems, the acceptor materials commonly exhibit a narrow bandgap with their absorption spectra extending to the near‐infrared region, which restricts the efficient harvesting of photons in the 600–750 nm.^[^ ^32^, ^33^, ^34^ ^]^ Considering these, the wide‐bandgap acceptor material is a better choice as the third component, which could establish more complementary absorption with most binary system to enhance the light absorption capacity, thereby increasing the short‐circuit current density (*J*_sc_). Additionally, the third component could form a cascade energy level alignment with the binary system to reduce energy loss and modulate the active layer morphology, then improving open‐circuit voltage (*V*_oc_), promoting favorable phase separation and reducing charge recombination. However, the selection and design of the third component with wide bandgap acceptors remain challenges, especially for its compatibility and interactions with the host acceptor materials.^[^ ^26^, ^35^ ^]^ In a ternary system, the strong intermolecular interactions and excellent miscibility between the two acceptors are essential for forming a uniformly mixed phase, which could favorably turn the phase separation and vertical phase distribution, optimize the morphology, and ultimately facilitate efficient exciton dissociation, charge transport and collection.^[^ ^36^, ^37^, ^38^, ^39^ ^]^ Therefore, designing wide‐bandgap acceptor materials with strong interactions and excellent miscibility with the host acceptor is essential for achieving high‐performance ternary OSCs.

In this study, we proposed an effective approach for high‐performance ternary OSCs by designing and synthesizing a cyano‐modified wide bandgap acceptor (named UF‐BCN) as the third component. The cyano (C≡N) group is a widely used substituent celebrated for its potent electron‐withdrawing capacity and high substituent constants. Hence, the CN group always serves as a linear electron‐withdrawing unit capable of i) polarizing π‐conjugated systems, ii) inducing intramolecular charge transfer (ICT) when connected to an electron‐donating unit, and iii) forming electron‐rich regions within the molecule.^[^ ^40^, ^41^, ^42^, ^43^, ^44^ ^]^ Additionally, the high polarity and rigid structure of the CN group could enhance the molecular interactions and packing abilities, which are pivotal for optimizing the morphology of active layers.^[^ ^45^ ^]^ Despite these, the modification of the CN groups in the central unit of SMAs has rarely been investigated, leaving a gap in understanding their impact on material properties and device performance.^[^ ^40^ ^]^ Against this backdrop, the non‐fused acceptor UF‐BCN was designed and synthesized with a cyano‐functionalized central unit on the benzene ring of the control molecule (named UF‐B). The incorporation of the CN group on the central unit not only elevates the surface energy but also substantially deepens the HOMO energy level, leading to a blue‐shifted absorption spectrum due to the electron‐withdrawing effect of CN group, rendering UF‐BCN an ideal candidate for ternary OSCs, and the non‐fused acceptors could reduce the cost of the materials in OSCs.^[^ ^46^, ^47^ ^]^ When introduced as the third component in the D18:BTP‐eC9 system, UF‐BCN exhibits complementary absorption and forms stronger intermolecular interactions and excellent miscibility with the acceptor BTP‐eC9, resulting in favorable phase separation and vertical phase distribution, which are conducive to efficient charge transport and reduced recombination. Consequently, the ternary OSC based on D18:BTP‐eC9:UF‐BCN achieves a remarkable PCE of 19.34%. This study provides a new insight into the molecular engineering of wide bandgap SMAs and paves the way for further advancements in high‐efficiency ternary OSCs.

## 2 Results and Discussion

**Figure 1:**
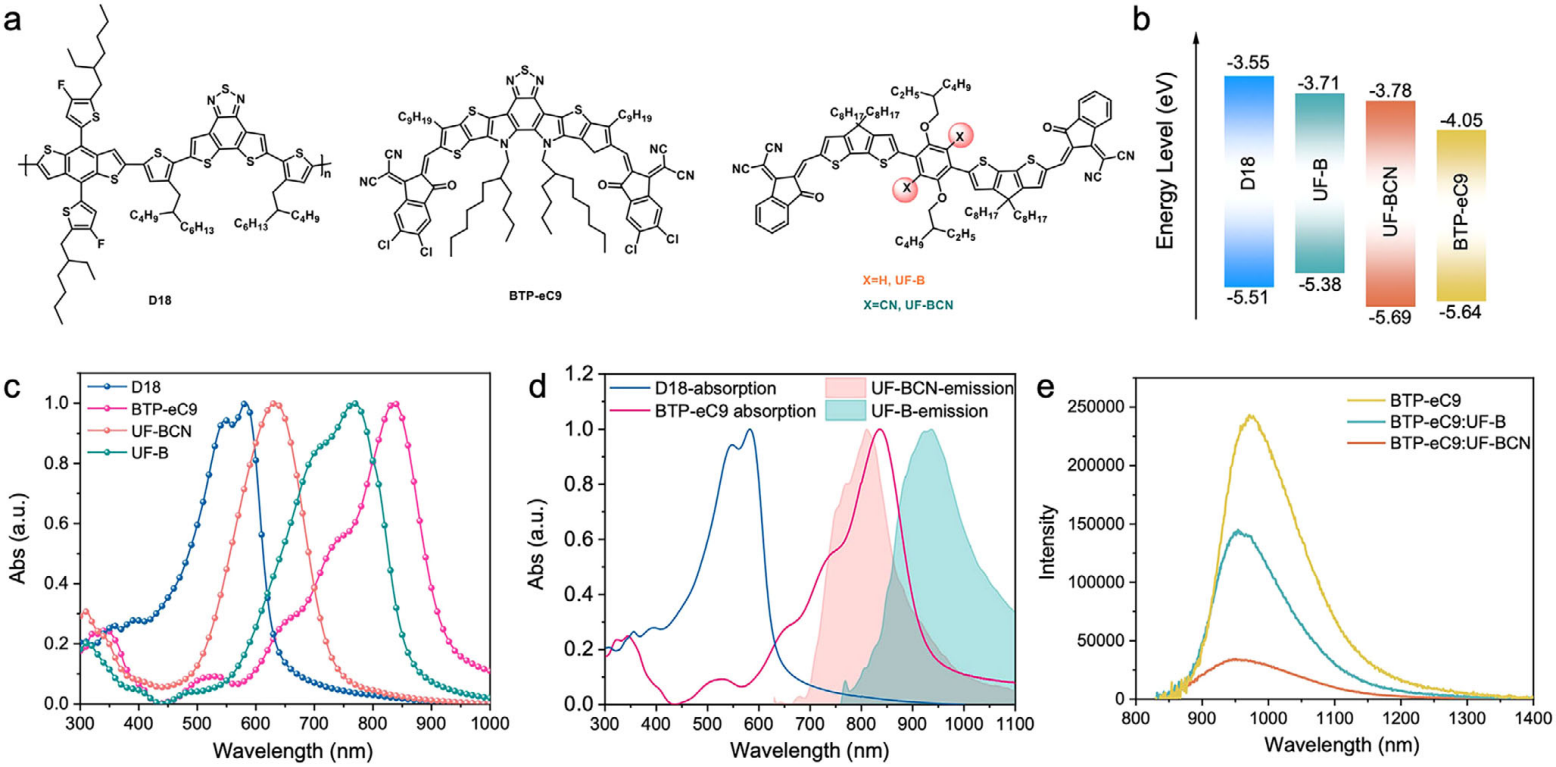
a) Molecular structures of D18, BTP‐eC9, UF‐B and UF‐BCN. b) Energy levels diagrams of D18, BTP‐eC9, UF‐B and UF‐BCN. c) The normalized absorption spectra of D18, BTP‐eC9, UF‐B and UF‐BCN in neat films. d) The PL spectra of UF‐B and UF‐BCN together with the absorption spectra of D18 and BTP‐eC9 in films. e) The PL spectra of BTP‐eC9, BTP‐eC9:UF‐B and BTP‐eC9:UF‐BCN films.

The chemical structures of D18, BTP‐eC9, UF‐BCN, and the control UF‐B were illustrated in **Figure** **1**a and the synthesis routes and characterization of UF‐B and UF‐BCN were provided in the Supporting Information. The thermal stability of UF‐B and UF‐BCN was investigated by the thermogravimetric analysis (TGA) (Figure S1, Supporting Information), and both exhibited good thermal stability with 5% weight loss temperatures exceeding 300 °C. Cyclic voltammetry (CV) measurements were carried out to explore the electrochemical properties as depicted in Figure 1b and Figure S2 (Supporting Information). The highest occupied molecular orbital (HOMO) and the lowest unoccupied molecular orbital (LUMO) levels of UF‐B and UF‐BCN were determined to be −5.38/−3.71 eV and −5.69/−3.78 eV, respectively, which are in excellent accordance with the calculated values by density functional theory (Figure S3, Supporting Information). Evidently, the introduction of CN groups in UF‐BCN induces a substantial downshift of the HOMO level and a cascade energy alignment establishing among the active layer materials, which could effectively facilitate charge dissociation and transport, thus reduce energy losses and improving the fill factor (*FF*) in the devices. Furthermore, the absorption spectra disclosed a prominent blue‐shifted onset for UF‐BCN compared to UF‐B (Figure 1c), indicating a much larger optical band gap (*E*_g_) of UF‐BCN (1.66 eV) than that of UF‐B (1.40 eV, **Table** **1**). The film absorption spectra of UF‐B and UF‐BCN along with the D18 and BTP‐eC9 were also presented in Figure 1c. It is clearly that there is a distinct main overlapping absorption region between UF‐B and BTP‐eC9. Conversely, UF‐BCN exhibits greater complementary with the D18:BTP‐eC9 system, which could help to broaden the light absorption range and improve the *J*_sc_ of the ternary OSCs. Besides, the maximal absorption coefficients of UF‐BCN in chloroform solution are much higher than that of UF‐B (Figure S4, Supporting Information). The detailed parameters are summarized in Table 1.

**Table 1:** Optical and Electrochemical Data of UF‐B and UF‐BCN.

Comp.	λ_max_^sol^ [nm]	λ_max_^film^ [nm]	λ_oneset_ [nm]^a^ ^)^	ε_max_ [M^−1^ cm^−1^]^b)^	E_g_ [eV]^c^ ^)^	HOMO [eV]	LUMO [eV]
UF‐B	725	768	884	1.40×10^5^	1.40	−5.38	−3.71
UF‐BCN	622	633	745	1.67×10^5^	1.66	−5.69	−3.78

^a)^ Absorption oneset of the molecule films;

^b)^ Molar extinction coefficient at λmax in solution;

^c)^ Optical bandgap was obtained from the onset wavelength of the molecule film, *E*_g_ = 1240/ λ_onset._

To gain deeper insights into the working mechanisms of UF‐B and UF‐BCN in the ternary OSCs, the photoluminescence (PL) spectra of UF‐B and UF‐BCN films were measured. As illustrated in Figure 1d, the emission spectra of UF‐BCN partially overlapped with the absorption spectra of BTP‐eC9, while those of UF‐B exhibits less overlapping, implying the potential for energy transfer from UF‐BCN to the acceptor BTP‐eC9 in line with the Förster theory.^[^ ^48^ ^]^ Additionally, the PL spectra of BTP‐eC9, BTP‐eC9:UF‐B and BTP‐eC9:UF‐BCN films were also obtained. As depicted in Figure 1e, when UF‐BCN or UF‐B was blended with BTP‐eC9, the PL spectra of the blend films was markedly changed and the quenching efficiency was 44.76% for BTP‐eC9:UF‐B and 86.69% for BTP‐eC9:UF‐BCN, indicating a much more efficient charge transfer process between BTP‐eC9 and UF‐BCN.

**Figure 2:**
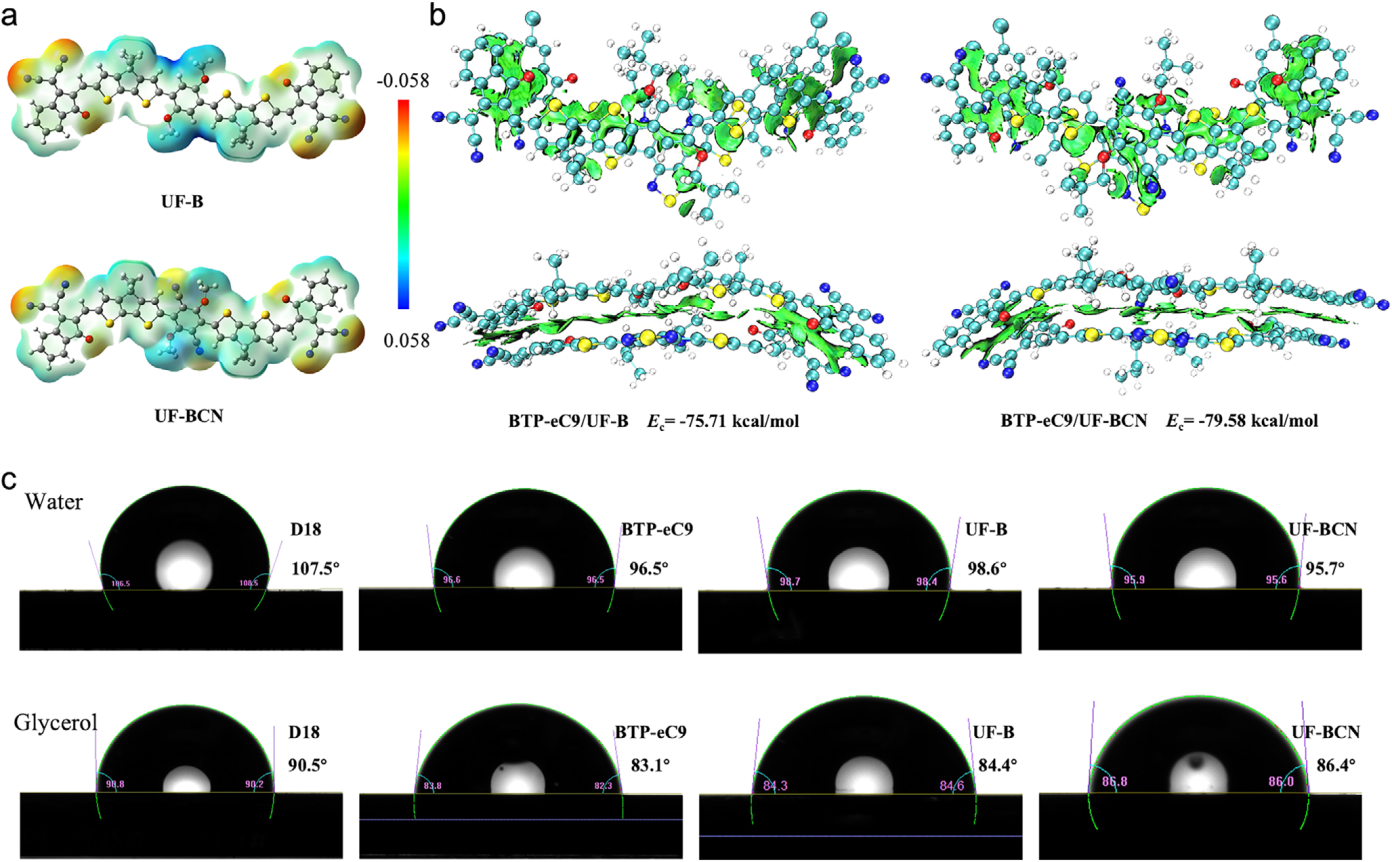
a) The electrostatic potential of UF‐B and UF‐BCN. b) Graphic representation and molecular energy of intermolecular interactions for BTP‐eC9/UF‐B and BTP‐eC9/UF‐BCN at side‐ and front‐view. c) Contact angles of D18, BTP‐eC9, UF‐B, and UF‐BCN thin films by applying deionized water and glycerol liquid drops.

To investigate the intermolecular interactions between the molecules, the electronic static potential (ESP) of UF‐B and UF‐BCN were calculated. As depicted in **Figure** **2**a and Figure S5 (Supporting Information), upon the CN group modification, the central unit of UF‐BCN displays a more negative electrostatic surface potential compared to that of the UF‐B. In contrast, the BTP‐eC9 exhibits a predominantly positive ESP distribution across the central unit. Therefore, based on the principle of opposite polarity attraction, it can be concluded that there is a strong intermolecular interaction between UF‐BCN and BTP‐eC9. To precisely quantify the interactions between UF‐B or UF‐BCN and BTP‐eC9, their complexation energies (*E*_c_) were computed using density functional theory (DFT), as shown in Figure 2b. The results reveal that UF‐BCN:BTP‐eC9 complex exhibits a higher *E*_c_ value of −79.58 Kcal mol^−1^ compared to −75.71 Kcal mol^−1^ for UF‐B:BTP‐eC9 complex, suggesting the BTP‐eC9 prefers to assemble with UF‐BCN and establishes a more favorable transport pathway in the D18:BTP‐eC9:UF‐BCN system. To further explore the influence of the chemical structure of materials on the surface free energy (*γ*), the contact angle of water and glycerol on the four neat films were measured as exhibited in Figure 2c. The *γ* values can be calculated by Owensn‐Wendt‐Kaelble's model and the obtained values are 27.76, 38.91, 36.68 and 37.19 mN m^−1^ for D18, BTP‐eC9, UF‐B and UF‐BCN, respectively. Compared with UF‐B, UF‐BCN has a larger *γ* value due to the introduction of the hydrophobic CN group. Additionally, the miscibility between the materials was further investigated based on the Flory−Huggins interaction parameter (*χ*) using the *γ* values of each single component.^[^ ^48^ ^]^ The calculated *χ* values of D18:BTP‐eC9, D18:UF‐B, D18:UF‐BCN are 0.939 *K*, 0.620 *K* and 0.688 *K*, respectively, where *K* is a constant. For the BTP‐eC9:UF‐B and BTP‐eC9:UF‐BCN, the *χ* values are 0.033K and 0.019K, respectively, indicating the excellent miscibility of BTP‐eC9 and UF‐BCN, and benefiting for the formation of a homogeneous phase, which could enhance the phase separation and are crucial for efficient exciton dissociation and charge extraction.

**Figure 3:**
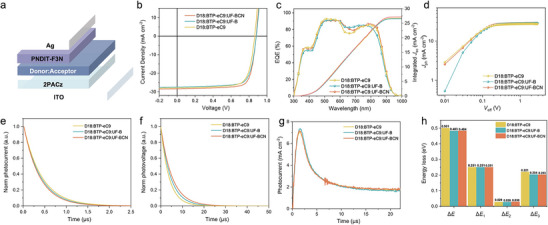
a) Diagram of a conventional device structure. b) *J‐V* curves. c) EQE curves. d) *J*_ph_‐*V*_eff_ curves. e) Transient photocurrent measurements. f) Transient photovoltage measurements. g) Photo‐CELIV curves of the devices based on D18:BTP‐eC9, D18:BTP‐eC9:UF‐B, and D18:BTP‐eC9:UF‐BCN. h) Comparison of *E*_loss_ for the binary and ternary devices.

The devices with or without the third component were fabricated with a conventional device architecture of ITO/2PACz/active layers/PNDIT‐F3N/Ag as shown in **Figure** **3**a. The detailed fabrication processes of the OSCs are provided in the Supporting Information. The current versus voltage (*J‐* *V*) curves of the optimized devices are presented in Figure 3b, and the corresponding optimized photovoltaic parameters are listed in **Table** **2**. Compared to the device based on D18:BTP‐eC9, the device incorporating D18:BTP‐eC9:UF‐B as the ternary system exhibits an increased *V*_oc_ of 0.881 V and improved *FF* of 75.60%, thereby elevating the efficiency from 18.03% to 18.29%. Meanwhile, the device with UF‐BCN as the third component achieves a higher efficiency of 19.34%, mainly ascribed to the further enhanced *FF* of 77.52% and *J*_sc_ of 28.36 mA cm^−2^. The external quantum efficiencies (EQEs) of all devices are shown in Figure 3c. Based on the EQE spectral response, the integrated *J*_sc_ for the D18:BTP‐eC9, D18:BTP‐eC9:UF‐B and D18:BTP‐eC9:UF‐BCN devices are 26.76, 26.59 and 27.16 mA cm^−2^, respectively, which are in good agreement with the *J*_sc_ values in the *J*–*V* curves. Compared to the binary device, the ternary devices exhibit significantly improved response in the short wavelength range of 600–700 nm, indicating that the complementary absorption of UF‐B and UF‐BCN with the D18:BTP‐eC9 blend film. However, the ternary system of D18:BTP‐eC9:UF‐B exhibits a weakened response in the range of 800–850 nm compared to the binary system, while the D18:BTP‐eC9:UF‐BCN shows no obvious change, furtherly demonstrating UF‐BCN could well mixed with BTP‐eC9.

**Table 2:** The photovoltaic parameters of the binary and ternary based OSCs.

Active layer	*V*_oc_ [V]	*FF* [%]	*J*_sc_ [mA cm^−2^]	PCE [%]^a^ ^)^
D18:BTP‐eC9	0.863	74.96	27.87	18.03 (17.83 ± 0.14)
D18:BTP‐eC9:UF‐B	0.881	75.60	27.46	18.29 (18.02 ± 0.17)
D18:BTP‐eC9:UF‐BCN	0.880	77.52	28.36	19.34 (18.98 ± 0.16)

^a)^ The average values in parentheses are obtained from 10 independent cells.

To further explore the impact of cyano‐modified UF‐BCN on charge generation and transport in active layers, the photocurrent (*J*_ph_) was measured as a function of the effective applied voltage (*V*_eff_) for the binary and ternary devices. Here, *J*_ph_ = *J*_L_‐*J*_D_, where *J*_L_ and *J*_D_ are the current densities under illumination and in the dark, respectively. Moreover, *V*_eff_ = *V*_0_‐*V*_appl_, where *V*_0_ is the voltage at which *J*_L_ = *J*_D_, and *V*_appl_ is the applied bias voltage. When the *V*_appl_ is sufficiently high (>2 V), all excitons disassociated into free carriers, and *J*_ph_ reaches a saturation state (*J*_sat_). Thus, the efficiency of exciton dissociation (*η*_diss_) or charge collection (*η*_coll_) can be defined as the ratio of *J*_ph_ to *J_sat_* values under short‐circuit conditions or maximum power output conditions, respectively. As shown in Figure 3d, the *η*_diss_ and *η*_coll_ values of the D18:BTP‐eC9, D18:BTP‐eC9:UF‐B and D18:BTP‐eC9:UF‐BCN devices are estimated to be 0.984,0.988, 0.996, and 0.830, 0.872, 0.895, respectively. Evidently, employing UF‐BCN as the third component can effectively improve exciton dissociation efficiency of the OSCs.^[^ ^49^ ^]^ Meanwhile, the transient photovoltage and photocurrent (TPV and TPC) measurements were conducted to investigate the charge carrier dynamics of the devices. As shown in Figure 3e, D18:BTP‐eC9:UF‐BCN device revealed a sweepout time of 0.37 *µs*, which is much shorter than those of the D18:BTP‐eC9 and D18:BTP‐eC9:UF‐B devices (0.41 and 0.39 *µs*), suggesting that the D18:BTP‐eC9:UF‐BCN device could effectively facilitate charge carrier extraction. For the TPV measurement results (Figure 3f), the carrier lifetimes are 3.76, 4.52, and 5.20 µs for the D18:BTP‐eC9, D18:BTP‐eC9:UF‐B, and D18:BTP‐eC9:UF‐BCN devices, respectively. The longer carrier lifetime of D18:BTP‐eC9:UF‐BCN device support its weaker recombination and higher *FF*.^[^ ^50^ ^]^ These findings aligned with the observed improvements in *FF* and overall device performance, highlighting the crucial role of cyano group in the molecular design for the third component with the ternary system.

The electron mobility (*µ*_e_) and hole mobility (*µ*_h_) of the devices were measured by the space‐charge‐limited‐current (SCLC) method, and the fitting curves were presented in Figure S6 (Supporting Information). The hole and electron mobilities of the devices based on D18:BTP‐eC9, D18:BTP‐eC9:UF‐B, and D18:BTP‐eC9:UF‐BCN are 1.75×10^−4^/2.28×10^−4^, 1.68×10^−4^/2.17×10^−4^, and 2.31×10^−4^/2.59×10^−4^ cm^2^ V^−1^ s^−1^, respectively, corresponding to the *µ*_h_/*µ*_e_ values of 1.30, 1.29 and 1.12. It was observed that both the hole and electron mobilities in the D18:BTP‐eC9:UF‐BCN devices were higher than those in other devices. Notably, the most balanced *µ*_h_/*µ*_e_ of the D18:BTP‐eC9:UF‐BCN system contributed to its highest *FF*.^[^ ^51^, ^52^ ^]^ Additionally, the photoinduced charge‐carrier extraction at linearly increasing voltage (photo‐CELIV) measurements were further performed to evaluate the effect of cyano‐modified UF‐BCN as the third component on the charge mobility of the ternary devices.^[^ ^39^ ^]^ In Figure 3g, the OSCs based on the D18:BTP‐eC9, D18:BTP‐eC9:UF‐B and D18:BTP‐eC9:UF‐BCN blend films showed photo‐CELIV mobilities of 2.18×10^−4^, 2.02×10^−4^ and 2.43×10^−4^ cm^2^ V^−1^ s^−1^, respectively. The cyano‐modified UF‐BCN based ternary system yielded highest photo‐CELIV mobility, which is beneficial for enhancing charge transport, and facilitating the achievement of increased *J*_sc_ and *FF* values.

In view of the *V*_oc_ of the ternary device was also enhanced concurrently, the detailed *E*_loss_ analysis of the optimized binary and ternary devices were carried out according to the literature method.^[^ ^53^, ^54^ ^]^ As shown in Figure 3h, the ternary device based on D18:BTP‐eC9:UF‐B and D18:BTP‐eC9:UF‐BCN exhibited lower *E*_loss_ with values of 0.483 eV and 0.484 eV, respectively, compared to the binary device with a value of 0.501 eV, which is arised from the lower non‐radiative energy loss (q∆*V*_non‐rad_) in the ternary systems. The optimal D18:BTP‐eC9:UF‐B and D18:BTP‐eC9:UF‐BCN ternary system yields higher electroluminescence quantum efficiency (EQE_EL_) of 3.77×10^−4^ and 3.91×10^−4^ than the binary system of 1.94×10^−4^ as shown in Figure S7 (Supporting Information), corresponding to the calculated ∆*V*_non‐rad_ of 0.204, 0.203, and 0.221 V, respectively. The results demonstrated that the ternary strategy is an effective approach to suppress the nonradiative energy loss, thereby benefiting for the improvement of *V*_oc_ in OSCs.

**Figure 4:**
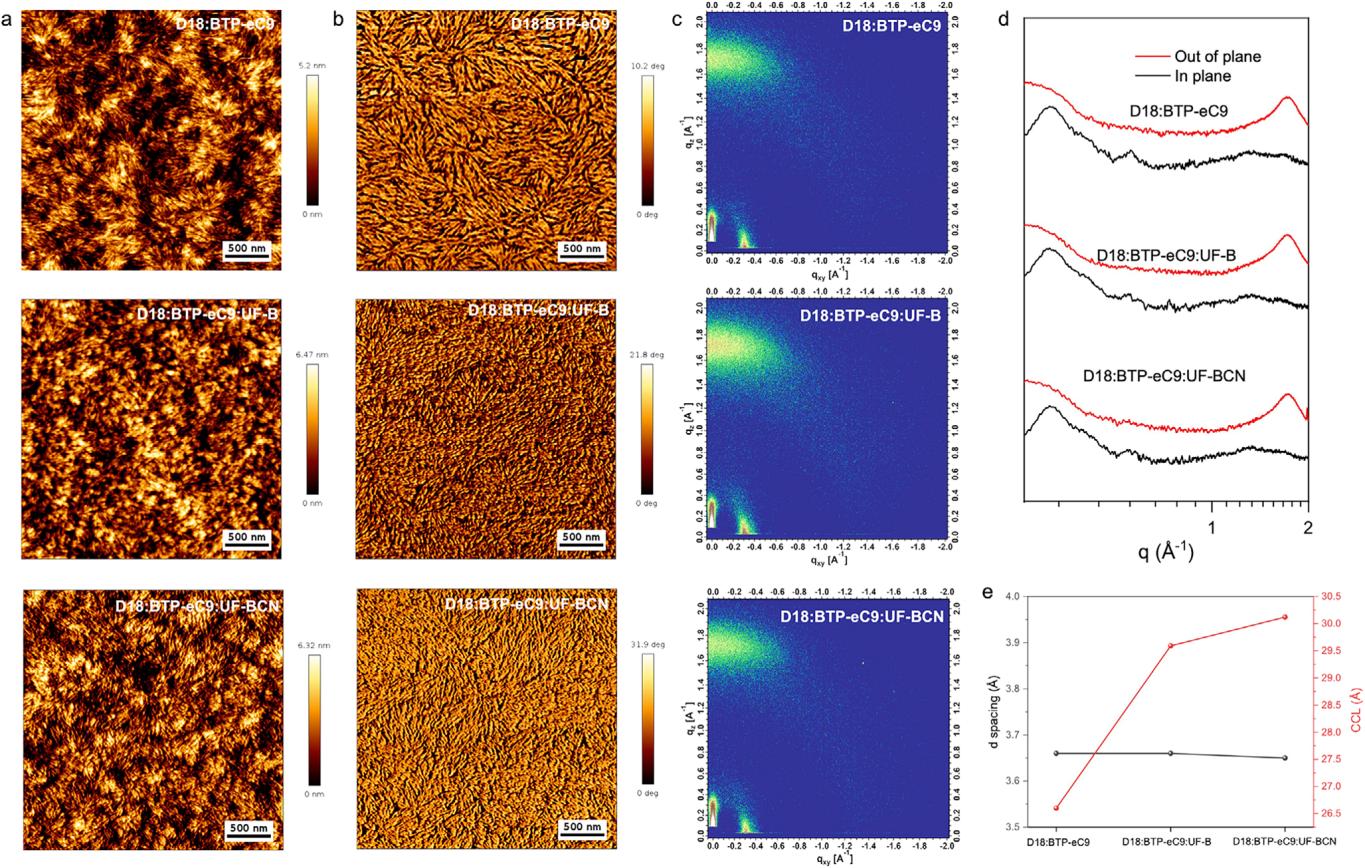
a) AFM height images. b) AFM phase images. c) 2D GIWAXS patterns. d) 1D OOP and IP line‐cut profiles of D18:BTP‐eC9, D18:BTP‐eC9:UF‐B, and D18:BTP‐eC9:UF‐BCN films. e) d‐spacings and CCL values for the corresponding GIWAXS results.

Furthermore, the atomic force microscopy (AFM) was employed to elucidate the impact of cyano‐modified UF‐BCN as the third component on the surface morphologies of the blend films. As shown in **Figure** **4**a,b, the addition of cyano‐modified UF‐BCN to the D18:BTP‐eC9 blend film (with a root‐mean‐square roughness (RMS) of 1.19 nm) led to a much better fibrous‐network structure accompanied by slightly increased RMS values of 1.47 nm, then effectively promoting the exciton dissociation and charge transportation,^[^ ^55^, ^56^ ^]^ which may be related to its strong interaction and excellent miscibility with the host components. The AFM phase images further reveal interpenetrating structure with smaller fibers in the two ternary blend films, particularly in the D18:BTP‐eC9:UF‐BCN ternary system, which is beneficial for the charge dissociation and transport, then endowing higher *FF* of the corresponding devices. To gain deeper insight into molecular stacking and the influence of the introduction of the third components of cyano‐modified UF‐BCN as the third component, the Grazing incidence wide‐angle X‐ray scattering (GIWAXS) was conducted. As depicted in Figure 4c–e, the binary and ternary blend films of D18:BTP‐eC9, D18:BTP‐eC9:UF‐B and D18:BTP‐eC9:UF‐BCN exhibited strong diffraction peaks in the out‐of‐plane (OOP) direction ≈1.72 Å^−1^, corresponding to π‐π stacking distances (d_π‐π_) of 3.66, 3.66, and 3.65 Å, respectively, and demonstrating a dominant face‐on orientation of the molecular stacking of these systems. Then, to further quantify the relative crystallinity levels, the crystal coherence lengths (CCLs) of the π−π stacking peaks of the three blends were calculated. As indicated in Figure 4e, the CCL values of the π−π stacking peak are 26.60, 29.59, and 30.12 Å for D18:BTP‐eC9, D18:BTP‐eC9:UF‐B, and D18:BTP‐eC9:UF‐BCN films, respectively. Notably, UF‐BCN based ternary system demonstrated a smaller π‐π stacking distances and rather larger CCL values compared to the binary system and ternary system based on UF‐B, which facilitated efficient charge transport and suppressed recombination losses, ultimately contribute to the superior *FF* and device performance.

**Figure 5:**
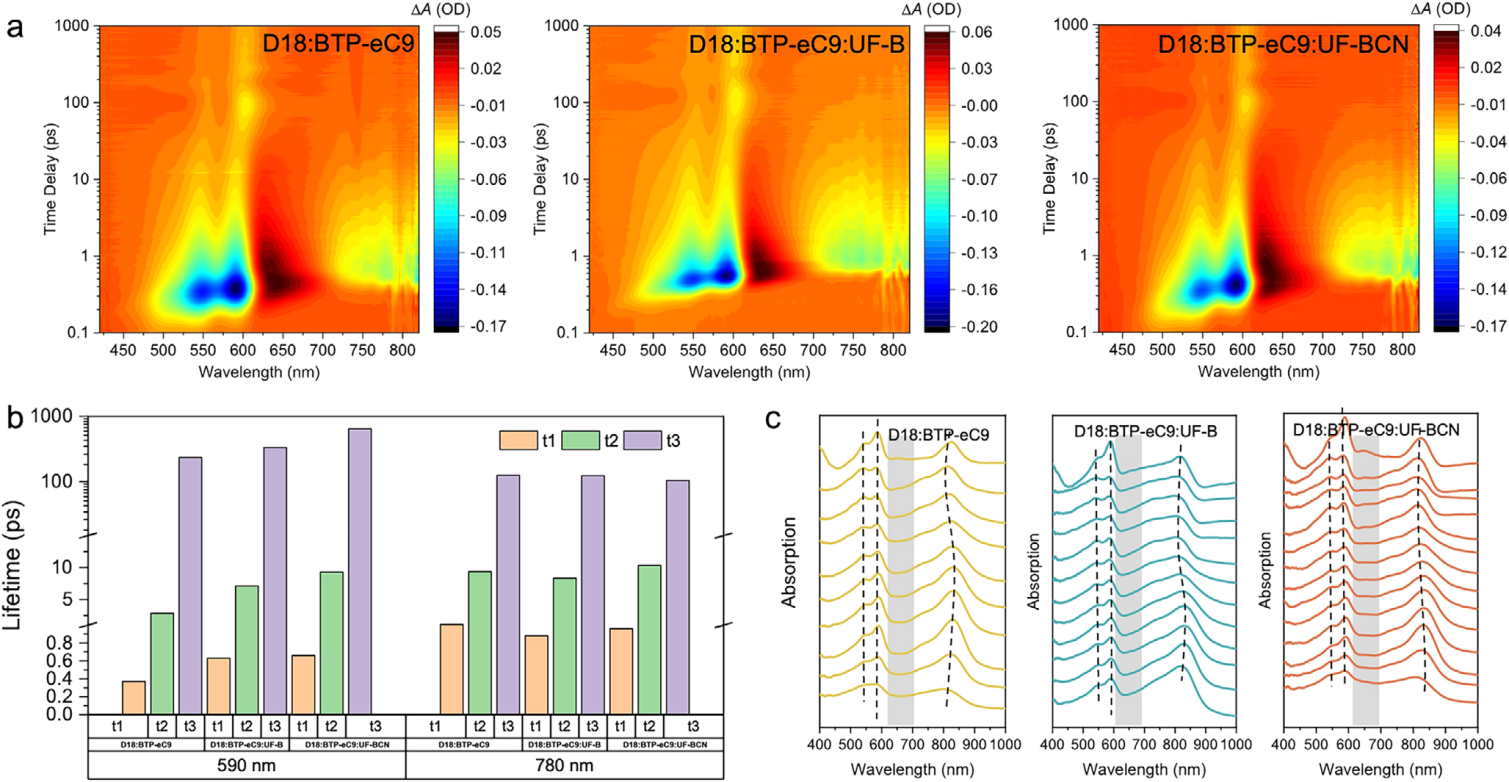
a) Contour map of fs‐TA results of D18:BTP‐eC9, D18:BTP‐eC9:UF‐B, and D18:BTP‐eC9:UF‐BCN devices. b) Exciton lifetimes of D18:BTP‐eC9, D18:BTP‐eC9:UF‐B, and D18:BTP‐eC9:UF‐BCN devices extracted from GSB signals at 590 and 780 nm for D18 and BTP‐eC9, respectively. c) Film‐depth‐dependent light absorption spectroscopy of binary films and ternary films spin coated on the ITO/2PACz substrate.

The femtosecond transient absorption spectrum (fs‐TAS) was employed to investigate the photophysical processes in the ternary blends incorporating UF‐B or UF‐BCN as the third components.^[^ ^57^ ^]^ As shown in the **Figures** **5**a and S8 (Supporting Information), the negative signals observed at ≈590 nm and ≈780 nm correspond to the ground state bleaching (GSB) signals of D18 and BTP‐eC9, respectively, and the positive signals at ≈633 nm originates from the excited state absorption (ESA). According to the triexponential function, three lifetimes (τ) can be consequently yielded for each decay, among which τ_1_ reflects the ultrafast exciton dissociation at the donor/acceptor interface, τ_2_ corresponds to the exciton diffusion time prior dissociation, and τ_3_ represents the dynamics of free charges.^[^ ^57^ ^]^ The τ_1_ values for the binary and ternary system at both 590 and 780 nm were comparable, indicating similar ultrafast hole/electron transfer at the interfaces. However, the D18:BTP‐eC9:UF‐BCN ternary blend exhibited prolonged τ_2_ values at both wavelengths, suggesting enhanced exciton diffusion lengths that suppress charge recombination and contribute to improve *J*_sc_ and *FF*. Additionally, compared to the D18:BTP‐eC9 and D18:BTP‐eC9:UF‐B system, the slightly larger τ_3_ observed in the D18:BTP‐eC9:UF‐BCN system implies suppressed geminate recombination at the interface and enhanced charge collection efficiency. The film‐depth‐dependent light absorption spectroscopy (FLAS) measurement was further conducted to semi‐empirically assess the kinetically and vertically resolved miscibility and vertical phase separation with the corresponding blends as illustrated in Figure 5c. The D18:BTP‐eC9:UF‐BCN ternary films exhibited shallower absorption valleys (less sharp) than those of the corresponding binary blends and D18:BTP‐eC9:UF‐B ternary film, indicative of a homogeneous distribution of UF‐BCN across the film depth direction.^[^ ^58^ ^]^ Furthermore, the characteristic absorption peaks of D18 and BTP‐ec9 in the D18:BTP‐eC9:UF‐BCN ternary blend films demonstrated better consistency across different film‐depths, reflecting reduced energy disorder, which could help to preserve the integrity of the charge transport channels, further rationalizing the superior device performance.^[^ ^59^, ^60^ ^]^

## 3 Conclusion

In this work, we demonstrated the significant potential of cyano substitution in the central unit for designing the wide bandgap small molecule acceptor to enhance the efficiencies of ternary OSCs. By incorporating the cyano group into the central unit, the newly designed acceptor, UF‐BCN, exhibited enhanced surface energy, a deeper HOMO energy level, and a blue‐shifted absorption spectrum compared to the control molecule UF‐B. When introduced as the third component into the D18:BTP‐eC9 system, UF‐BCN demonstrated complementary light absorption, strong intermolecular interactions, and excellent miscibility with BTP‐eC9, which facilitated the formation of a well‐mixed acceptor phase, and then enabled UF‐BCN to effectively optimize the phase separation and vertical phase distribution, promote the charge transport and reduce the non‐radiative voltage loss of the ternary system. Consequently, the D18:BTP‐eC9:UF‐BCN based ternary OSC achieved an impressive PCE of 19.34%. Our study highlights the role of cyano substitution in central unit engineering and offers a strategic approach to developing high‐performance ternary OSCs.

## Conflict of Interest

The authors declare no conflict of interest.

## Supporting information



Supporting Information

## Data Availability

The data that support the findings of this study are available in the supplementary material of this article.
