# Supplementary material for: Modulated Interactions Induced by Cyano‐Modified Wide‐Bandgap Small‐Molecule Acceptors Enables High‐Performance Ternary Organic Photovoltaics
Source: Adv Sci (Weinh). 2025 Jun 20;12(34):e06606. doi: 10.1002/advs.202506606 (PMC12442590; doi:10.1002/advs.202506606)
Supplement: Supplementary file 1 — Supporting Information [file ADVS-12-e06606-s001.docx]

Supporting Information

Modulated Interactions Induced by Cyano-Modified Wide-bandgap Small-Molecule Acceptors Enables High-Performance Ternary Organic Photovoltaics

Yuanyuan Zhang^†^, Shijie Cheng^†^, Meijia Chang*, Lei Wang, Huanhuan Gao, Zirui Wang, Guanghao Lu, Shengmin Gan, Xinbo Lv, Jin Wang, Qingqing Sun, Mingjun Niu, Zichao Shen, Zhijun Wu, Cao Yang*, Xuying Liu, Lingxian Meng*

**1. Materials and synthesis**

All chemicals were purchased from commercial sources and used without further purification unless stated otherwise. BTP-eC9 was purchased from Organtec Ltd. D18 was purchased from Hyper photoelectric Ltd. ITO glass was purchased from were purchased from Liao Ning RightChoice Tech Ltd.

**Scheme S1.** Synthetic routes of small molecular acceptor (UF-BCN).

The synthesis steps of UF-BCN are as follows:

**Synthesis of Compound 2*.*** A solution of compound 1 (1.00 g, 2.03 mmol) and CuCN (85 mg, 6.09 mmol) in dry N,N-Dimethylformamide (DMF, 25 mL) were degassed three times in a 100 mL two-necked flask. After being stirred at 145 ℃ for 48h, 500 mL dilute ammonia water (15%) was added to remove the excess CuCN. Then, the mixture was extracted with CHCl_3_ (50 mL × 3). The organic layer was combined and washed with water, dried with anhydrous Na_2_SO_4_. After removal of solvent, the crude product was purified by silica gel using petroleum ether as eluent to give the compound 2 as a brownish-yellow solid. (56% yield).

**Synthesis of Compound 3.** To a mixture of compound 2 (0.58 g, 1.57 mmol) and N-bromosuccinimide (0.81 g, 4.71 mmol) in 100 mL rounded flask, trifluoroacetic acid (12 mL) was added until the above reactant was completely dissolved. Then, concentrated sulfuric acid (3 mL) was added and the mixture was stirred 2h at room temperature. The solution was neutralized with the addition of saturated sodium carbonate solution, and then extracted with CH_2_Cl_2_. The combined organic layer was washed with water and brine, dried over Na_2_SO_4_. After removal of solvent, the crude product was purified by silica gel using petroleum ether as eluant to afford compound 3 as a white solid. (73% yield)

**Synthesis of Compound 4.** A solution of compound 3 (0.60 g, 1.51 mmol) and (4,4-bis(2-ethylhexyl)-4H-cyclopenta[2,1-b:3,4-b']dithiophene-2-yl)tributyltin (2.61 g, 3.78 mmol) in toluene was degassed three times followed by the addition of Pd(PPh_3_)_4_ (0.26 g, 0.23 mmol) as catalyst in to a 100 mL two-necked flask. After stirring and refluxing under the protection of argon for 24h, the mixture was extracted with CHCl_3_ (50 mL × 3). The organic layer was combined and washed with water, dried with anhydrous Na_2_SO_4_. After removal of solvent, the crude product was purified by silica gel using dichloromethane and petroleum as eluent to give the compound 4 as an orange solid. (44% yield)

**Synthesis of UF-BCN-CHO.** A Vilsmeier reagent, which was prepared with POCl_3_ (5.13 g, 33.50 mmol) in dry DMF (8 mL), was added to a solution of compound 3 (0.80 g, 0.67 mmol) in 1,2-dichloroethane (100 mL) at 0 ℃ and then stirred at room temperature for 2h. After being stirred at 75 ℃ for 12h, the mixture was poured into ice water (200 mL), neutralized with NaOAc, and then extracted with dichloromethane. The combined organic layer was washed with water and brine, dried over Na_2_SO_4_. After removal of solvent, the crude product was purified by silica gel using a mixture of dichloromethane and petroleum ether as eluant to afford UF-BCN-CHO as a red solid (84% yield).

**Synthesis of UF-BCN.** UF-BCN-CHO (0.10 g, 0.076 mmol) and IN (0.80 g, 0.45 mmol) were dissolved in CHCl_3_ (20 mL) and pyridine (0.7 mL) was added dropwise. Under the protection of argon, the mixture was stirring overnight at room temperature. After removal of solvent, the crude product was purified by silica gel using chloroform and petroleum as eluant to afford the target material UF-BCN as dark blue solid. (78% yield)

^1^H NMR (400 MHz, CDCl_3_) δ 8.99 (s, 2H), 8.73 (d, J = 7.4 Hz, 2H), 8.01 – 7.90 (m, 2H), 7.87 – 7.56 (m, 8H), 3.81 (d, J = 4.0 Hz, 4H), 2.11 – 2.00 (m, 7H), 1.93 – 1.83 (m, 2H), 1.13 – 0.92 (m, 31H), 0.87 (t, J = 5.6 Hz, 11H), 0.80 – 0.75 (m, 11H), 0.66 (t, J = 7.0 Hz, 11H).

^13^C NMR (75 MHz, CDCl_3_) δ 215.43, 188.57, 163.35, 159.82, 156.56, 139.97, 139.64, 137.51, 137.38, 136.90, 135.05, 134.37, 128f.43, 125.47, 125.27, 123.67, 121.40, 114.82, 114.13, 100.01, 99.99, 54.37, 40.23, 35.55, 34.11, 29.82, 28.80, 28.57, 28.30, 27.39, 22.99, 22.80, 14.07, 10.93, 10.66, 10.30.

The synthesis routes of UF-B are displayed in Scheme S2, and the same procedures of UF-BCN were used. The corresponding data of UF-B is summarized below:

**Scheme S2.** Synthetic routes of small molecular acceptor (UF-B).

**UF-B** (80% yield)

^1^H NMR (400 MHz, CDCl_3_) δ 8.93 (s, 2H), 8.67 (d, J = 7.2 Hz, 2H), 7.90 (d, J = 6.1 Hz, 2H), 7.78 – 7.62 (m, 6H), 7.55 (s, 2H), 7.29 (s, 2H), 4.11 (d, J = 4.4 Hz, 4H), 2.00 (d, J = 4.9 Hz, 10H), 1.72 – 1.53 (m, 10H), 1.41 (s, 8H), 1.13 – 0.87 (m, 46H), 0.80 – 0.60 (m, 28H).

^13^C NMR (75 MHz, CDCl_3_) δ 215.43, 202.01, 182.04, 165.13, 140.01, 138.90, 138.05, 136.85, 134.68, 134.00, 125.07, 123.61, 115.29, 112.56, 111.99, 111.32, 100.00, 83.79, 66.99, 60.79, 53.78, 43.41, 35.42, 34.20, 33.98, 30.73, 29.20, 28.43, 27.27, 24.21, 23.12, 22.80, 16.35, 14.21, 14.09, 11.24, 10.63.

**2. Devices Fabrication and Characterization**

OSCs were fabricated with the device configuration of ITO/2PACz/active layer/PNDIT-F3N/Ag. Before using, patterned ITO glasses were cleaned via sequential sonication in detergent, de-ionized and isopropanol and then blow-dried by nitrogen. All pre-cleaned ITO substrates were treated by oxygen plasma for 15 minutes to improve their work function and clearance. The pre-treated ITO substrates were coated with 2PACz (0.3 mg mL^-1^ in Ethanol) by spin-coating (3000 rpm. for 30 s) and then baked at 100℃ on a hotplate for 10-15 min in air. Then the 2PACz-coated ITO substrates were transferred into a N_2_-filled glove box for subsequent steps. The D18:BTP-eC9 (1:1 w/w) was dissolved in chloroform at the donor concentration of 4 mg mL^-1^ with 4 mg mL^-1^ solid additive of 1,3,5-tribromobenzene (TBB). D18:BTP-eC9:UF-B and D18:BTP-eC9:UF-BCN (1:1.2:0.2 w/w/w) was dissolved in chloroform at the donor concentration of 4 mg mL^-1^ (with 4 mg mL^-1^ TBB as additive). All the active layers were spin-coated at 2000 rpm for 30 s. Then thermally annealed at 80℃ for 5 min. The interfacial layer of PNDIT-F3N solution was deposited by spin-coating onto the active layers at 3000 rpm for 30 s. PNDIT-F3N was dissolved in methanol with the addition of 0.5 vol% acetic acid to prepare a 1 mg/ml solution. Finally, 120 nm Ag layer was deposited by thermal evaporation through a shadow mask under the vacuum of 3×10^-4^ Pa conditions. The device area is approximately 4.0 mm^2^, which was defined by the overlapping area of ITO anode and Ag cathode.

***J*-*V* and EQE Measurement**

The current-voltage (*J*-*V*) curves of all devices were measured in a high-purity nitrogen-filled glove box. AM 1.5G irradiation at 100 mW cm^-2^ was provided by the solar simulator (LSS-55, Shanghai Jinzhu Technology Co Ltd), which was calibrated by standard silicon solar cells (LRC-KG2). The external quantum efficiency (EQE) data were obtained using the solar-cell spectral-response measurement system (LST-QE, Shanghai Jinzhu Technology Co Ltd).

**Thermogravimetric (TGA)**

Thermogravimetric analysis (TGA) plots were measured with a Netzsch series instrument under a nitrogen atmosphere at heating and cooling rates of 10 ℃ min^-1.^

**Transient photocurrent (TPC) and photovoltage (TPV)**

TPC and TPV measurements were obtained on a Molex 180081-4320 with light intensity about 0.5 sun, Voltage and current dynamics were recorded on a digital oscilloscope (Tektronix MDO4104C). Voltages at open circuit and currents under short circuit conditions were measured over a 1 MΩ and a 50 Ω resistor, respectively.

**Electrochemical characterizations**

Cyclic voltammogram (CV) was performed with a LK2010 Microcomputer based Electrochemical Analyzer at a scan rate of 100 mV s^-1.^. The highest occupied molecular orbital (HOMO) and lowest unoccupied molecular orbital (LUMO) energy levels were calculated from the onset oxidation potential and the onset reduction potential, using the equation:

$E_{HOMO}=-\left( 4.8+E_{OX}^{onset} \right),E_{LUMO}=-\left( 4.8+E_{re}^{onset} \right)$.

**Optical characterizations**

The UV–vis spectra in dilute chloroform solution and films were tested on Agilent Technologies Cary 5000 spectrophotometer. All film samples were spin–cast on quartz substrates under the same conditions as those used for device fabrication.

**Electroluminescence and photoluminescence measurement**

Electroluminescence (EL) and photoluminescence (PL) spectra were taken using a Kymera-328I spectrograph and an EMCCD purchased from Andor Technology (DU970P). Injection current used for EL was 1 mA cm^-2^, and excitation wavelength used for the PL measurements was 500 nm.

**EQE_EL_ measurements**

EQE_EL_ measurements were done using a home-built setup using a Keithley 2400 to inject current to the solar cells. Emission photon-flux from the solar cells was recorded using a Si detector (Hamamatsu s1337-1010BQ) and a Keithley 6482 picoammeter.

**SCLC Measurement**

Hole and electron mobilities are evaluated by fabricating hole-only and electron-only devices with the device architectures of ITO/PEDOT:PSS/active layer/MoO_3_/Ag and ITO/ZnO/active layer/PDINN/Ag, respectively. And SCLC is described by:

$$J=\frac{9\varepsilon_{0}\varepsilon_{r}\mu_{0}{(V-V_{bi}）}^{2}}{8L^{3}}$$

where $J$ is the current density, $\varepsilon_{0}$ is the permittivity of free space (8.85$\times$10^-12^ F m^-1^), $\varepsilon_{r}$ is the relative dielectric constant of the transport medium, $\mu_{0}$ is the hole or electron mobility, $V$ is the applied voltage to the device, $V_{bi}$ is the built-in voltage due to the relative work function difference of the two electrodes and $L$ is the film thickness of the active layer.

**Density Functional Theory (DFT)**

Density functional theory calculations were conducted at the B3LYP/6-31 G* levels to obtain the optimized molecular geometries and frontier molecular orbitals of the acceptors.

**Calculation of Flory-Huggins interaction parameter (χ) by contact angle**

Contact angle measurements of each pure film were conducted utilizing deionized water and glycerol by drop shape analysis. The surface tension of each film was calculated via Wu’s model:

$$\gamma_{water}\left( 1+{cos\theta}_{water} \right)=\frac{4\gamma_{water}^{d}\gamma^{d}}{\gamma_{water}^{d}+\gamma^{d}}+\frac{4\gamma_{water}^{p}\gamma^{p}}{\gamma_{water}^{p}+\gamma^{p}}$$

$$\gamma_{glycerol}\left( 1+{cos\theta}_{glycerol} \right)=\frac{4\gamma_{glycerol}^{d}\gamma^{d}}{\gamma_{glycerol}^{d}+\gamma^{d}}+\frac{4\gamma_{glycerol}^{p}\gamma^{p}}{\gamma_{glycerol}^{p}+\gamma^{p}}$$

$$\gamma=\gamma^{d}+\gamma^{p}$$

where $\theta$ is the contact angle of each thin film, and $\gamma$ is the surface tension of samples, which is equal to the sun of the dispersion ($\gamma^{d}$) and polarity ($\gamma^{p}$) components; $\gamma_{water}$ and $\gamma_{glycerol}$ are the surface tensions of the water and glycerol; and $\gamma_{water}^{d}$, $\gamma_{glycerol}^{d}$, $\gamma_{water}^{p}$ and $\gamma_{glycerol}^{p}$ are the dispersion and polarity components of $\gamma_{water}$ and $\gamma_{glycerol}$. The molecular miscibility extent can be evaluated via Flory–Huggins interaction parameter $\chi$ determined through the follow formula:

$$\chi=K{(\sqrt{\gamma_{donor}}-\sqrt{\gamma_{acceptor}})}^{2}$$

**Atomic force microscope (AFM)**

AFM measurements were performed by using Bruker Dimension ICON in tapping mode. All film samples were spin-cast on glass/ITO/2PACz substrates.

**Grazing incidence wide angle X-ray scattering (****GIWAXS)**

GIWAXS was performed at MetalJet-D2, Excillum on the wavelength of 0.134144 nm with Xeuss 2.0. All simples were deposited on the silicon and were irradiated at a fixed X-ray incident angle of 0.2° with an exposure time of 1800 s.

**Film-depth-dependent light absorption spectra (FLAS) measurement**

The FLAS was acquired by a home-made in-situ instrument, and measured by using a spectrometer equipped with a soft plasma-ion source (PU100, Shaanxi Puguang Weishi Co. Ltd.). The power-supply for generating the soft ionic source is 100 W with input oxygen pressure ~10 Pa. Surface etching proceeded via a self-developed soft plasma technique, preserving the materials under the surface. And a spectrometer was used to in-situ monitor. FLAS is extracted from the evolution of the light absorption spectra during soft plasma etching. Film-depth-dependent absorption spectra were extracted by the evolution of the spectra and the Beer-Lambert's Law. A self-developed capacitive-coupled plasma generator was utilized to generate soft ionic plasma. This ensured the reliable test of the light absorption spectra of the films during etching.

**3. Supplementary Figures**


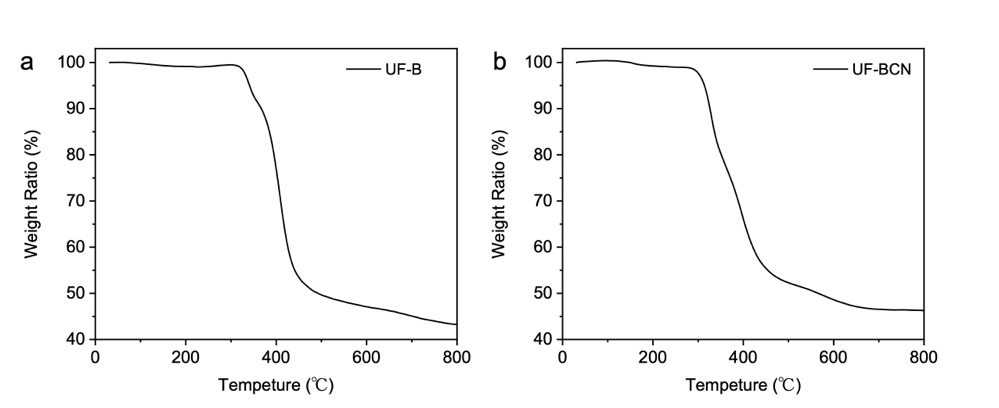


**Figure S1.** The thermogravimetric analysis (TGA) curves of (a) UF-B and (b) UF-BCN.


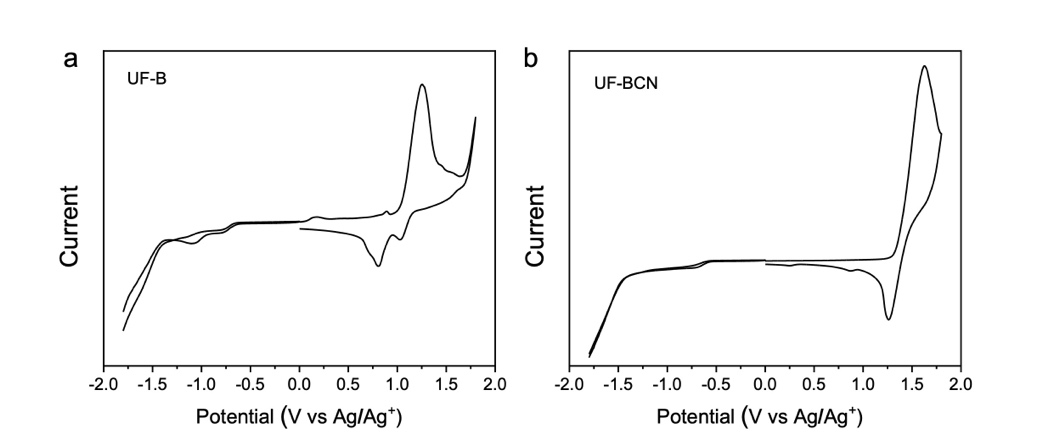


**Figure S2.** Cyclic voltammograms of (a) UF-B and (b) UF-BCN thin films in CH_3_CN solutions with 0.1 mol L^-1^ n-Bu_4_NPF_6_ at a scan rate of 50 mV s^-1^.


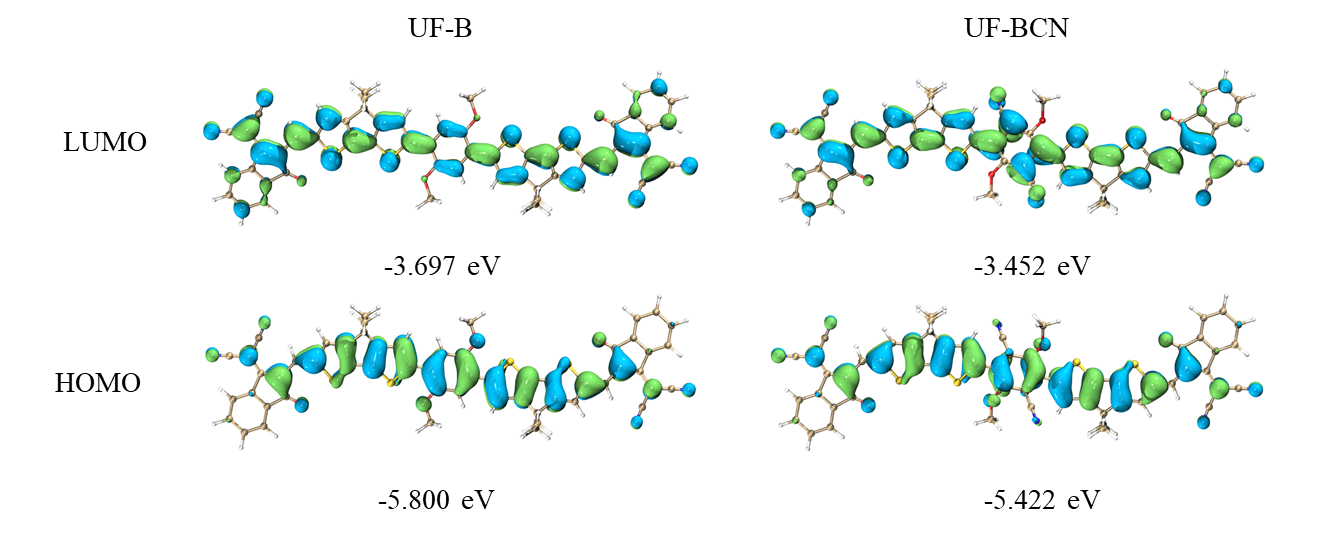


**Figure S3**. DFT-calculated frontier molecular orbitals with HOMO and LUMO energy levels of UF-B and UF-BCN.


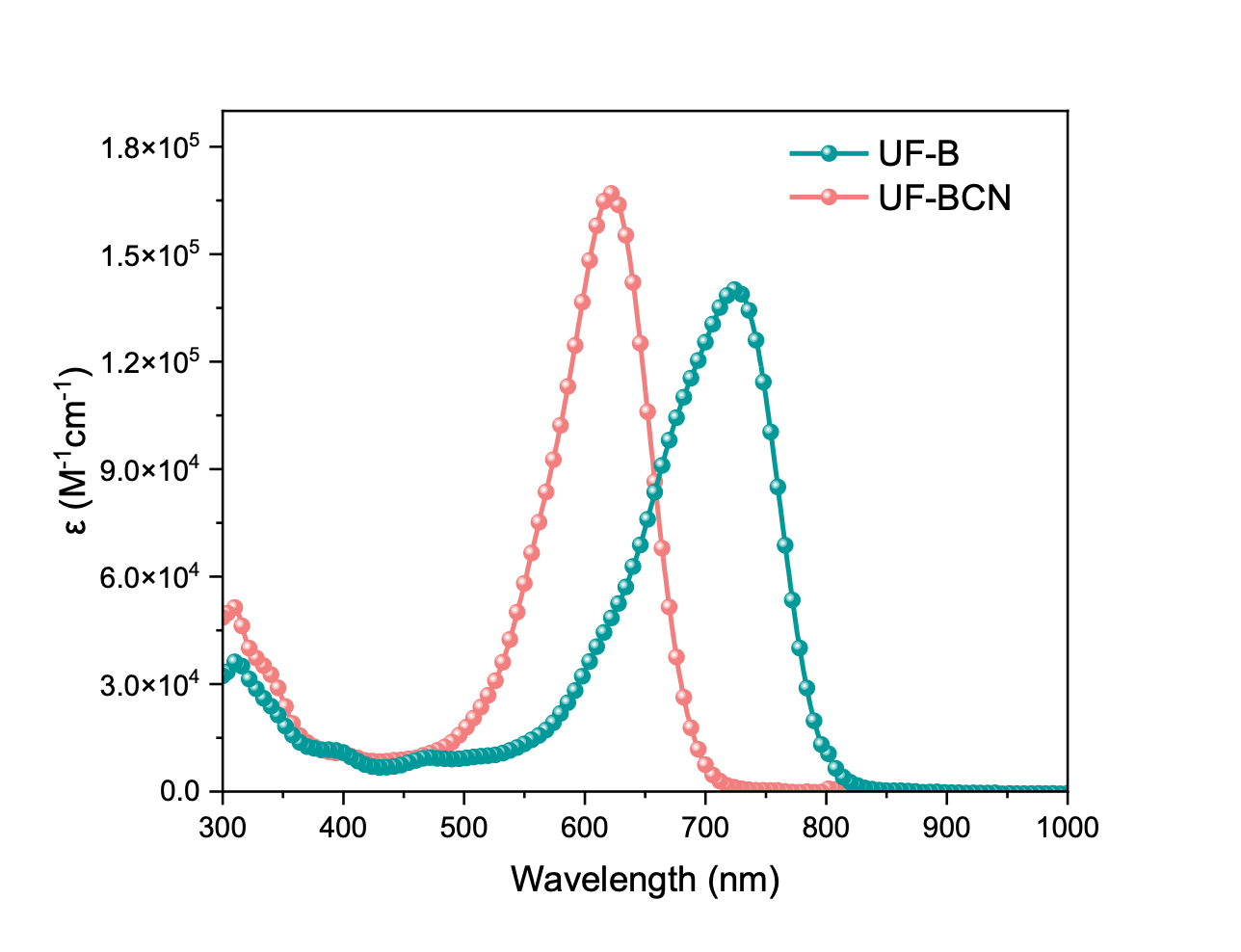


**Figure S4.** Absorption spectra of DTBTF in chloroform solution.


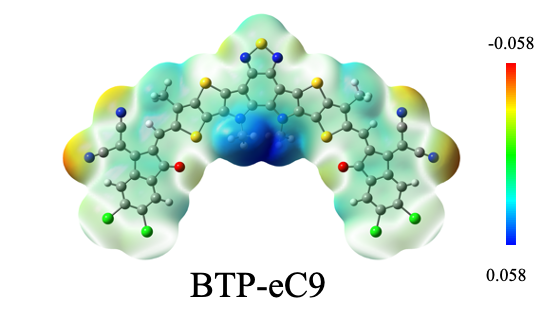


**Figure S5.** The electrostatic potential of BTP-eC9.


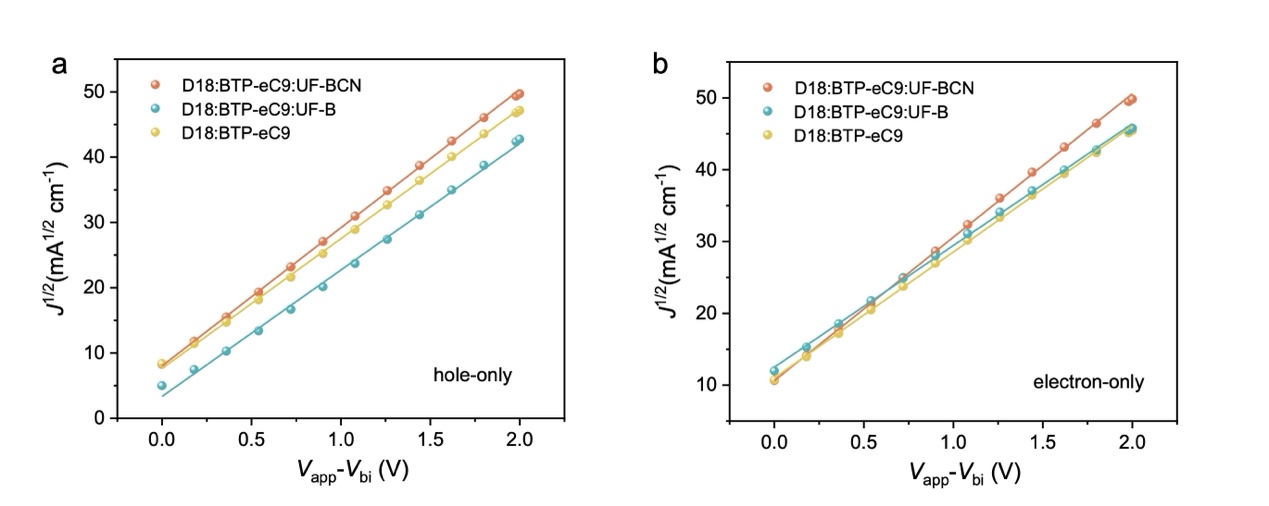


**Figure S6.** SCLC curves a) hole and b) electron of the devices based on D18:BTP-eC9, D18:BTP-eC9:UF-B and D18:BTP-eC9:UF-BCN.


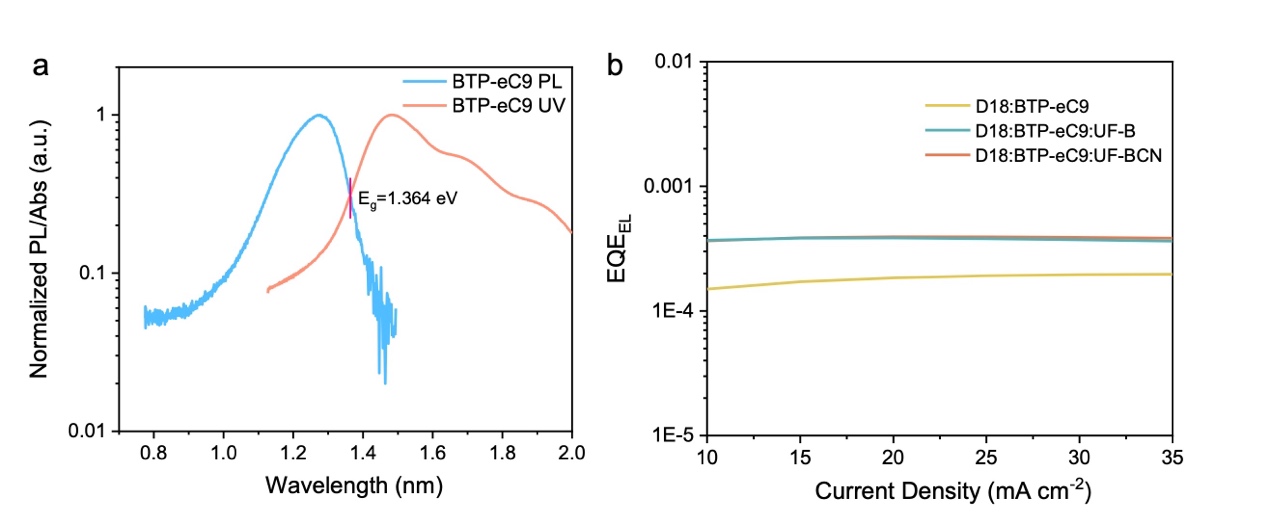


**Figure S7.** a) Normalized PL and UV-vis absorption of NFA BTP-eC9, *E*_g_^opt^ of D18:BTP-eC9, D18:BTP-eC9:UF-B and D18:BTP-eC9:UF-BCN blends are estimated by intersection point of PL and UV-vis for BTP-eC9. b) Normalized EQE_EL_ of the devices based on D18:BTP-eC9, D18:BTP-eC9:UF-B and D18:BTP-eC9:UF-BCN.


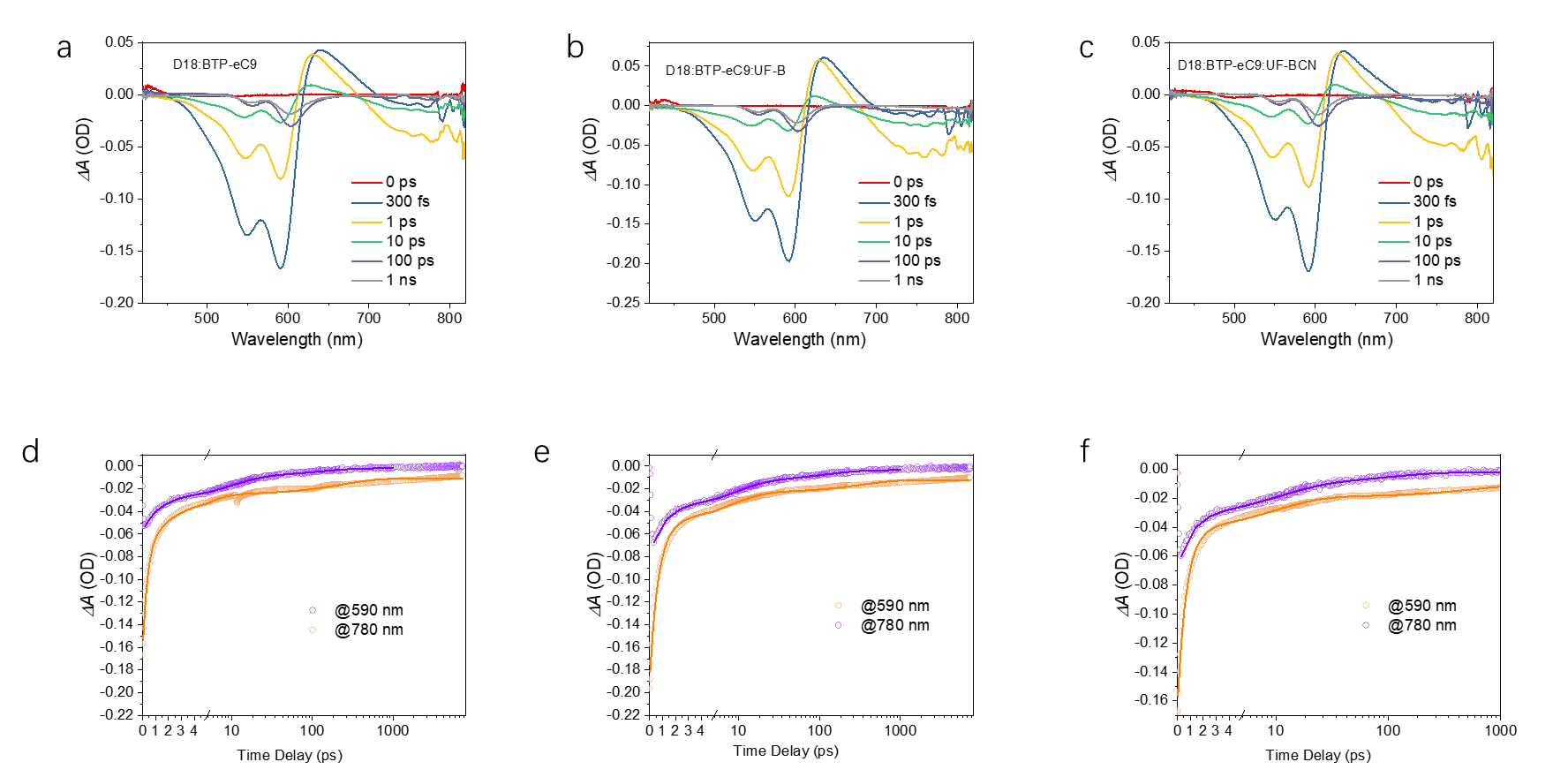


**Figure S8**. a-c) corresponding TA spectra recorded at different time delays. d-f) Ns-resolved decay dynamics probed at 590 and 780 nm of binary and ternary films.


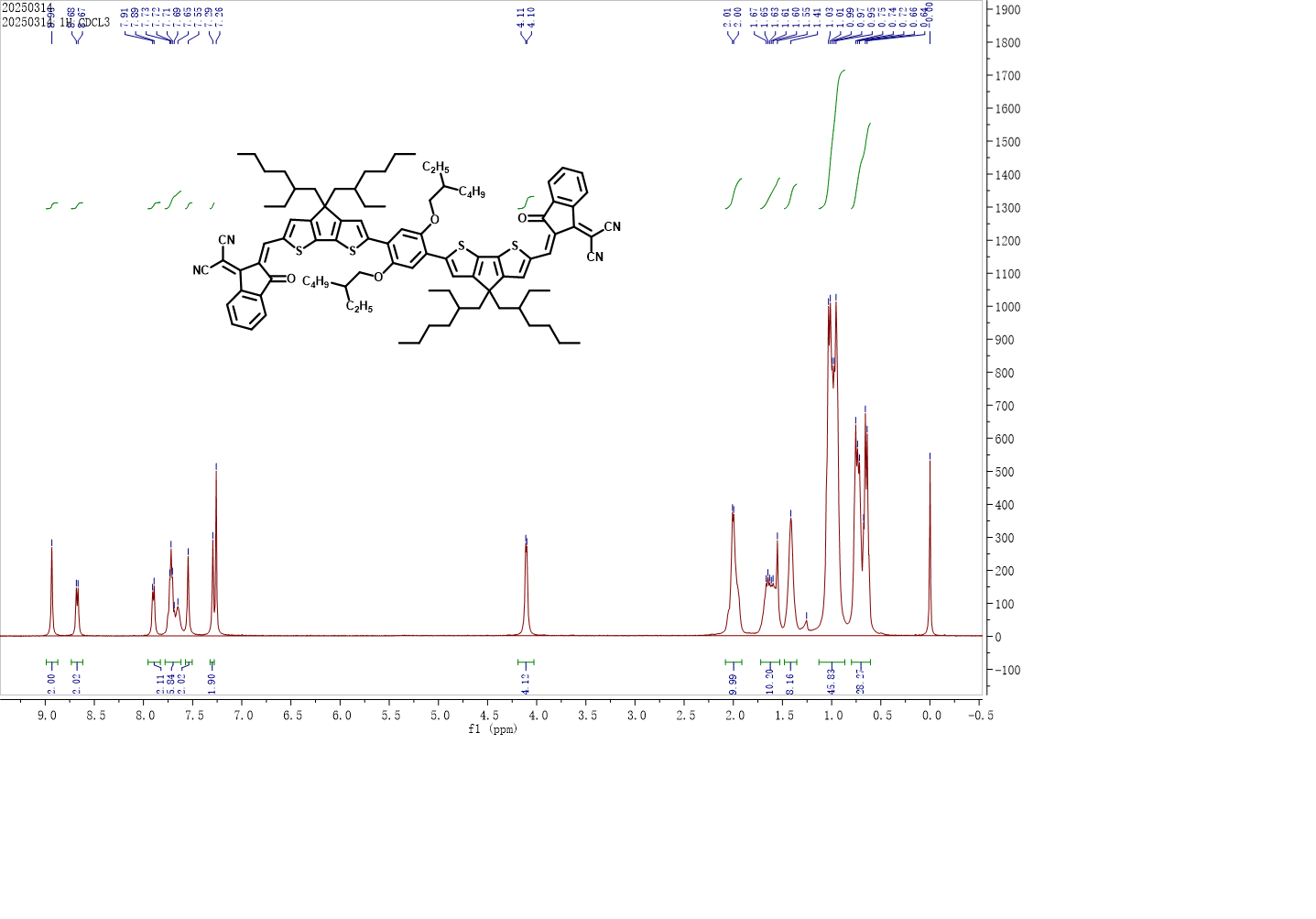


**Figure S9** ^1^H NMR of UF-B.


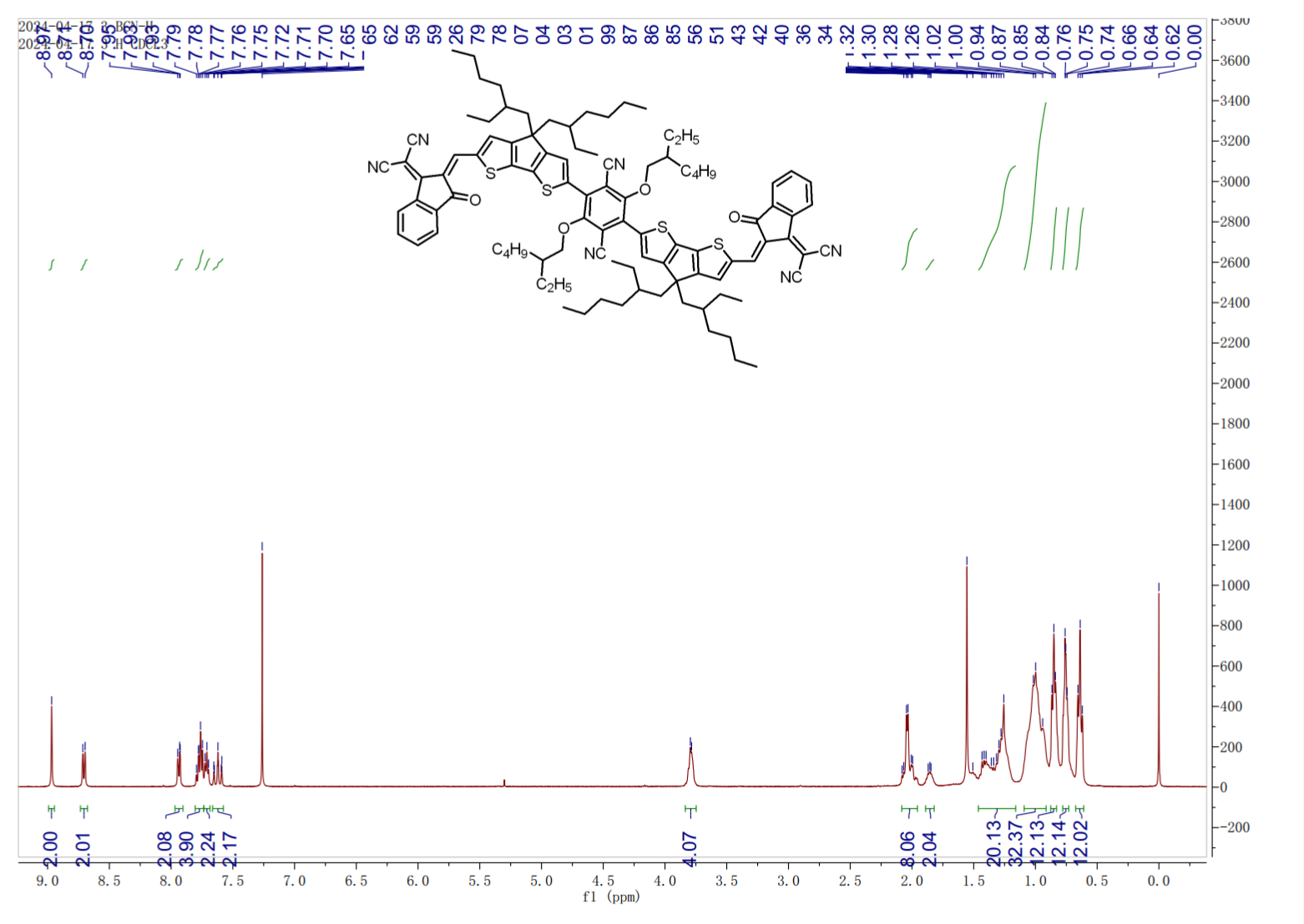


**Figure S10** ^1^H NMR of UF-BCN.

**Table S1.** Electron mobilities (*μ*_e_) and hole mobilities (*μ*_h_) of D18:BTP-eC9, D18:BTP-eC9:UF-B and D18:BTP-eC9:UF-BCN devices.

| Active layer | *μ*_h_ (cm^-2^ V^-1^ s^-1^) | *μ*_e_ (cm^-2^ V^-1^ s^-1^) | *μ*_h_/*μ*_e_ |
| --- | --- | --- | --- |
| D18:BTP-eC9 | 2.28×10^-4^ | 1.75×10^-4^ | 1.30 |
| D18:BTP-eC9:UF-B | 2.17×10^-4^ | 1.68×10^-4^ | 1.29 |
| D18:BTP-eC9:UF-BCN | 2.59×10^-4^ | 2.31×10^-4^ | 1.12 |

**Table S2.** The analysis data of the two-dimensional GIWAXS results for the blend films in the out of plane direction.

| Sample | 010 (OOP) | | | |
| --- | --- | --- | --- | --- |
|  | q (Å^-1^) | d (Å) | FWHM (Å^-1^) | Coherence length (Å) |
| D18:BTP-eC9 | 1.717 | 3.66 | 0.213 | 26.60 |
| D18:BTP-eC9:UF-B | 1.715 | 3.66 | 0.191 | 29.59 |
| D18:BTP-eC9:UF-BCN | 1.722 | 3.65 | 0.188 | 30.12 |
